# Evaluating the Effects of *Epichloë* Fungal Endophytes of Perennial Ryegrass on the Feeding Behaviour and Life History of *Rhopalosiphum padi*

**DOI:** 10.3390/insects15100744

**Published:** 2024-09-26

**Authors:** Nicholas Paul Collinson, Khageswor Giri, Jatinder Kaur, German Spangenberg, Mallik Malipatil, Ross Cameron Mann, Isabel Valenzuela

**Affiliations:** 1 Agriculture Victoria Research, AgriBio Centre for AgriBioscience, 5 Ring Road, Bundoora, VIC 3083, Australia; nick.collinson@agriculture.vic.gov.au (N.P.C.); khageswor.giri@agriculture.vic.gov.au (K.G.); jatinder.kaur@agriculture.vic.gov.au (J.K.);; 2 Department of Science, Health and Engineering, School of Applied Systems Biology, La Trobe University, Bundoora, VIC 3083, Australia; 3 College of Grassland Science, Qingdao Agricultural University, Qingdao 266000, China; germancspangenberg@gmail.com

**Keywords:** hemiptera–plant interactions, multitrophic interactions, electrical penetration graph, antibiosis, antixenosis, crop protection

## Abstract

**Simple Summary:**

Endophytic fungi in pasture grasses help the plant resist unfavourable biotic and abiotic conditions. Despite some well-documented aspects of how these fungi confer resistance to insects, some mechanisms are not well known. In this work, we measured the effects of such endophytes on aphids, an important pest group. We measured aphid feeding activity and life-history effects to further understand the mode of action of endophyte–perennial ryegrass systems. We were able to demonstrate that, in some cases, fungal endophytes significantly deterred feeding, but in other cases, feeding was not affected, and yet high mortality was observed. This suggests a complex scenario of interactions between compounds in the endophyte–perennial-ryegrass–aphid system. Our study shows that fungal endophytes have the potential to improve sustainable aphid control by decreasing the use of insecticide sprays.

**Abstract:**

The bird cherry-oat aphid, *Rhopalosiphum padi* (L.), is an economically significant pest of pasture grasses, the latter being capable of hosting several fungal endophyte–perennial ryegrass symbiota rich in alkaloids and toxic to vertebrates and invertebrates. Measuring aphid feeding behaviour can provide insights into the effectiveness and mode of action of different fungal endophytes. This study investigated the effects of different *Epichloë*–perennial ryegrass symbiota on the feeding behaviour of *R. padi* using the electrical penetration graph technique while also assessing the aphid life history. In most cases, endophytes had significant feeding deterrence and paired fecundity and mortality effects. But, in some instances, endophytes with the highest aphid mortality did not significantly deter feeding, suggesting a more complicated scenario of interactions between the relative concentration of metabolites, e.g., host plant defence response metabolites and alkaloids, and/or physical changes to leaf morphology. Overall, this study sheds light on the mode of action of *Epichloë* endophytes against aphids and highlights the importance of *Epichloë*–perennial ryegrass symbiota in the management of insect pests such as aphids in pasture-based grazing systems.

## 1. Introduction

Perennial ryegrass, *Lolium perenne* (L.), is native to Europe and is the most important perennial agricultural grass worldwide [1]. It is also the most commonly used pasture grass species in Australian and New Zealand dairy pastures [2,3]. It has several advantages over other pasture grasses, including strong persistence in fields for many years, high nutrient content and a level of resistance to drought and certain pests and diseases [1,2,3]. Despite these advantages, perennial ryegrass is still susceptible to biotic stresses, including some insect pests such as the Argentine stem weevil, *Listronotus bonariensis* (Kuschel) [4]; the African black beetle, *Heteronychus arator* (Fabricius) [5,6]; and several aphid species, including the bird cherry-oat aphid, *Rhopalosiphum padi* (L.) [7,8]. To defend against these stresses, perennial ryegrass has evolved a mutualistic relationship with fungal *Epichloë* endophytes such as *Epichloë festucae* var. *lolii*, which confers the host plant with not only further resistance to vertebrate and invertebrate herbivores but also a higher tolerance to drought and saline conditions [9]. Endophytes such as *Epichloë* protect plants from insect herbivory, predominantly through the production of bioactive alkaloids that can be toxic or unpalatable to insect pests [10,11]. The alkaloid peramine, in particular, is known for its strong feeding deterrent properties against the Argentine stem weevil, *L. bonariensis* [4,12]. Loline alkaloids, a different class of alkaloid compounds produced by *Epichloë* endophytes in fescue species, are known to have direct toxic insecticidal effects on *L. bonariensis* and the bird cherry-oat aphid, *R. padi* [13,14,15]. Indole diterpene alkaloids produced by *Epichloë* species are more commonly associated with neurotoxic effects on livestock; however, other indole diterpenes produced by other fungi have been demonstrated to have insecticidal activity (e.g., nodulisporic acids) [16]. The alkaloid ergovaline produced by many *Epichloë* species also has detrimental effects on grazing livestock, such as vasoconstriction and increased heat stress [17].

*Rhopalosiphum padi*, belonging to the Aphididae family of the Hemiptera order, is a common phloem-feeding pest of grains and pasture grasses [18]. It is often found in perennial ryegrass pasture-based grazing systems, where it can cause significant damage through direct feeding and the vectoring of viral plant diseases such as barley yellow dwarf virus (BYDV) [19]. In Australia, under laboratory conditions with adequate food sources like barley, a single female *R. padi* can produce a substantial number of offspring, typically between 50 and 80 nymphs [20]. This results in very rapid population growth under favourable climatic conditions when temperatures range from 20 to 25 °C [21]. Aphids such as *R. padi* feed by inserting anatomically adapted mouthparts (stylets) into plant tissues to extract phloem sap [22,23]. This feeding behaviour can be negatively affected by systemic insecticide treatments that deter aphids from feeding on the phloem [24]. Monitoring changes in aphid feeding behaviour can be an effective way to identify the mode of action of different insecticides.

One of the most effective methods for monitoring and quantifying changes in aphid feeding behaviour is the electrical penetration graph (EPG) technique [25]. The EPG technique was developed for the purpose of studying hemipteran feeding behaviour [26,27] and has since undergone continuous improvement to better understand the feeding behaviour of significant agricultural pests such as aphids [28]. This technique is used to study the movement of hemipterans’ stylets within the plant tissue through the creation of an electrical circuit between an electrode attached to the dorsum of the insect and an electrode inserted into the soil of the plant the insect is feeding on. Variations in the location of the stylet tip in the plant tissue result in fluctuations in electrical potential in the circuit, which are represented as different recognisable waveform patterns using both a detection device and specialised software [29]. Specific waveform patterns relate to different specific feeding and probing behaviours, such as phloem ingestion or xylem ingestion [28,30,31]. Therefore, changes in the electrical potential in the circuit can be monitored to assess stylet movements within plant tissue [23]. The use of the EPG allows for the quantification of the frequency and duration of aphid feeding behaviour on different host plants or when aphids are exposed to different treatments. This has practical uses in the study of insecticides, as insecticides that have feeding deterrence effects may result in waveforms showing fewer phloem ingestion events, or events of a shorter duration, in aphids feeding on insecticide-treated plants compared to aphids feeding on insecticide-free plants [32].

The main aim of this study was to investigate the effects of five different *Epichloë*–perennial ryegrass symbiota on *R. padi* feeding behaviour and life history. These treatments included four commercial endophytes (AR1, AR37, NEA2 and NEA6), as well as the wild type (standard endophyte, SE) and perennial ryegrass without endophytes—Nil_E. An additional aim of this study was to assess the effectiveness of the EPG technique for differentiating between endophyte treatments. In particular, the EPG technique has been applied to investigate endophyte effects on aphid feeding behaviour as part of a broader study aimed at determining effective methods for benchmarking novel *Epichloë* endophyte symbiota [33]. This technique could provide useful insights into the potential antifeedant mode of action of insecticidal fungal endophytes in perennial ryegrass. For instance, it could elucidate whether aphids are able to reach the vascular system and maintain sustained phloem ingestion. There is little specific research on the effects of endophytes on *R. padi* feeding behaviour using the EPG, and what research there is has mostly investigated only a single endophyte strain [34].

## 2. Materials and Methods

### 2.1. Aphids, Endophytes and Plants

*Rhopalosiphum padi* was collected in South-Eastern Australia (Horsham, Victoria) from oat, *Avena sativa* (L), in June 2009. To guarantee the absence of barley yellow dwarf virus (BYDV) in experimental populations, ten individuals of *R. padi* were isolated and cultivated on perennial ryegrass (*cv* Alto) seedlings for at least three generations. Nymphs were promptly transferred to new seedlings upon birth to mitigate any possible transmission of aphid-borne plant viruses [35]. Nymphs were removed and transferred using a fine paintbrush by gently touching the aphid until it withdrew its stylet and then picking it up with the paintbrush and placing it onto the new seedling. One clonal lineage was selected for further experiments. Colonies were maintained on perennial ryegrass in 24.5 × 24.5 × 63.0 cm BugDorm 42260F insect rearing cages with a mesh size of 150 × 150 µm (Megaview Science Co., Ltd., Taichung, Taiwan) at 20 ± 2 °C and 62.0 ± 5% RH and with a photoperiod of 14 h light–10 h dark.

To confirm aphid species identification, a molecular-based identification method was carried out using the barcode region of the *cytochrome oxidase subunit 1* (*CO1*) gene, following the same method as in previous work [33]. PCR products were sequenced by Macrogen Inc. (Seoul, Republic of Korea), and sequences were compared to public databases (NCBI BLASTn), determining 100% similarity and coverage with previously identified species. Sequences were submitted to GenBank (MT119781), and specimens preserved in both 70% and 100% ethanol were deposited in the Victorian Agricultural Invertebrate Collection (VAIC#086929 and VAIC#086930, respectively) at Agribio, Bundoora. Aphids were confirmed as *R. padi* (identification carried out by Valenzuela, I.) based on 100% sequence similarity with a previously sequenced population (DQ499057) from Australia [20].

Plants were germinated as per methods described in [33]. Perennial ryegrass seeds infected with fungal endophytes of the species *Epichloë festucae* var. *lolii* were sourced from Agriseeds (Christchurch, New Zealand). These included four different commercial endophyte varieties (AR1, AR37, NEA2 and NEA6), one wild type or standard endophyte (SE), which is found naturally in many perennial ryegrass pastures [36], and a control of perennial ryegrass without endophytes (Nil_E) (*cv* Alto) (Table 1). The aforementioned *Epichloë* endophytes produce a range of alkaloids [indole diterpenes (Lolitrem B and Janthitrem I, usually called epoxyjanthitrem), ergopeptides (Ergovaline) and pyrrolopyrazines (Peramine)], resulting in each endophyte having a unique alkaloid profile (Table 1). Studies have shown that endophytes are present and become metabolically active, i.e., produce alkaloids, from as early as 6 days after sowing and peak when seedlings are 8 to 10 days old [37,38]. In our study, the presence and identity of endophyte strains were confirmed using a Competitive Allele-Specific PCR (KASP) bioassay [39] on 150 (7-day-old) seedlings from the same batch of seeds used in the life-history and EPG assays, as per previous work [33]. The results from the KASP assay indicated a very high incidence of endophytes in each seed batch for all 5 endophyte symbiota (100% of seeds show endophyte presence) and null presence in perennial ryegrass without endophytes (Nil_E).

### 2.2. EPG Setup and Methodology

The electrical penetration graph (EPG) technique was used to monitor the feeding behaviour of adult *R. padi* on seven-day-old perennial ryegrass seedlings (one adult per plant) both with and without endophytes (SE, AR1, AR37, NEA2, NEA6 and Nil_E).

To create the circuit for EPG recordings, electrodes were constructed by attaching a 10–20 mm long, 18 µm thick gold wire to a 20 mm long copper wire using a water-based glue with suspended conductive silver particles. The copper wire was attached at the opposite end to the head of a copper nail using lead solder. The plant and the aphid were made part of an electrical circuit by first attaching the gold wire electrode to the aphid dorsum using the same conductive glue. This was accomplished by securing the aphid to a plastic pipette tip attached to a mild vacuum device (Javac Pty. Ltd., Rowville, Australia) and applying a small drop of the glue to the aphid dorsum and holding the tip of the gold wire in place until the glue cured. The first fully unfurled leaf of each seedling was affixed to a plastic tag using a metal clamp and foam padding to limit movement. The copper nail ends of the electrodes were connected to the EPG Giga8d device via the input of the head stage amplifier with a one-giga-ohm input resistance and 50 × gain (EPG Systems, Wageningen, The Netherlands). To complete the circuit, copper soil electrodes, 2 mm thick × 10 cm long copper rods, were inserted into the soil of seven-day-old perennial ryegrass seedlings and connected to the plant voltage output plug of the EPG device. Thus, the aphid inserting its stylet into the plant (probing) closed the circuit, and the resulting electrical signals were recorded.

We ran all eight electrodes in each EPG recording session, with one aphid and one seedling connected to each electrode for each treatment. The final number of EPG recordings ranged between 20 and 24 per treatment. Measurements were performed during the first 12 h of contact between aphids and the test plants. Experiments were conducted in a Faraday cage and in an environment maintained under laboratory conditions (20 ± 2 °C and constant lighting from standard fluorescent tubes).

### 2.3. EPG Waveforms and Variables

Seven different EPG waveforms have been identified relating to different aphid probing, feeding and non-probing activities [23]. For probing, C represents the first electrical stylet contact with the epidermis and intercellular sheath salivation in the epidermis and mesophyll; pd represents the drop in electrical potential observed when intracellular punctures by the stylet occur; and F represents stylet derailment, where the stylets become separated from each other, or there are other stylet penetration difficulties. For feeding, E1 represents the sieve element salivation directly prior to phloem ingestion, E2 represents phloem sap ingestion (i.e., feeding), and G represents the ingestion of xylem sap as opposed to phloem. For non-probing, Np represents the non-probing period when the stylet is withdrawn and probing is not taking place. Waveforms were recorded using the Stylet+d software (v 01.25), while the identification and analysis were conducted using the Stylet+a software package (v 01.25), and these were compared to proven examples representing different aphid feeding behaviours [23].

### 2.4. Life-History Bioassays

Bioassays were conducted to assess the *R. padi* life history on different endophyte treatments as per the methods in Collinson et al. [33]. In brief, perennial ryegrass (*cv* Alto) seeds with and without endophyte seeds were germinated on a Petri dish with moistened 90mm filter paper (Whatman^TM^) at room temperature (approximately 23 °C and 63% RH). After 7 days, seedlings were transferred into 4 mL clear plastic cups (Olympus Packaging, Pty. Ltd., Ferntree Gully, Australia), with a small piece of moistened cotton placed on top of the roots [33] to maintain their viability for 7 days. This process was repeated every 7 days for 4 weeks to allow for 4 weeks of life-history assessment (the experiment was based on 24 replicates of one 7–14-day-old seedling/aphid/week for 4 weeks). Apterate aphids reared on endophyte-free perennial ryegrass (Nil_E) were individually placed in 4 mL clear plastic cups (Olympus Packaging, Pty. Ltd., Ferntree Gully, Australia), each with a single Nil_E perennial ryegrass seedling, and monitored every day to collect new-born nymphs. Twenty-four new-born nymphs per treatment were collected using a fine paintbrush and placed individually into the plastic cups (experimental units) containing seven-day-old seedlings of endophyte-free (Nil_E) or endophyte-infected (SE, AR1, AR37, NEA2, NEA6) perennial ryegrass, as explained above. Each aphid was monitored every day and placed onto a new 7-day-old seedling every week for 4 weeks. The parameters investigated in this study were (a) fecundity: the number of nymphs born over a 28-day period; (b) mortality: life expectancy up to 28 days; and (c) the intrinsic rate of increase (*r_m_*). Fecundity was measured by counting the number of nymphs born every 24 h for 28 days from the moment of birth; mortality was measured by recording the number of aphids that died each day up to 28 days, and *r_m_* was calculated using the formula *r_m_* = 0.74(log_e_*M*_d_)/*d*, where *d* = the length of the pre-reproductive period, and *M*_d_ = the number of progeny produced in a period of time equal to *d* [40]. The aphid life history was monitored every day for 28 days, and aphids were transferred onto a new seedling every seven days. All experiments were carried out in a controlled environment room at 20 ± 2 °C and 62.0 ± 5% RH and with a photoperiod of 14 hr light–10 hr dark. Twenty-eight days was chosen as the duration of the assay, as it represents a major portion of the average reproductive lifespan of *R. padi* on barley at 20 °C [20,41].

### 2.5. Statistical Analyses

To analyse EPG data, aphid feeding behaviour was recorded using the Stylet+a software [23] and quantified by 102 separate metrics to define the seven major waveforms using a specialised quantification package in Microsoft Excel (usable under Microsoft Excel 98 and later versions) [42]. Numerical data categorised by these metrics were analysed using a one-way analysis of variance (ANOVA). Differences between treatment means were examined using the least significant difference (LSD) at a 5% level of significance. The unit of analysis was individual aphids connected to EPG electrodes (24 replicates per treatment). Residuals-versus-fitted-values plots were examined to determine any need for data transformation to ensure the normality of residuals with constant variance. Prior to the final analyses, the feeding behaviour data were logarithmically transformed to satisfy the assumption of normality with constant variance.

For the aphid life-history analysis, *R. padi* nymph mortality was grouped into four time periods: 0–24 (24 h), 24–48 (48 h), 48–72 (72 h) and >72 (>72 h) hours. Adult mortality was grouped into three time periods: 14, 21 and 28 days. Daily observed fecundity data were grouped into three time periods: 14, 21 and 28 days. Fecundity and *r_m_* were both calculated using only aphids that survived to reproduction. The difference in the mortality of aphid nymphs and adults between treatments at each time period was analysed using logistic regression models, where the number of aphid deaths at each time period was the response variable (success), and logit was the link function. The Wald chi-squared test was used to include/exclude a term in/from the model. Data on fecundity and *r_m_* were analysed using a one-way ANOVA. Differences between treatment means were examined using the least significant difference (LSD) at a 5% level of significance. The unit of analysis was ‘individual aphids in plastic cups’ (24 replicates). Residuals-versus-fitted-values plots were examined to determine any need for data transformation to ensure the normality of residuals with constant variance. All data in this study were analysed using GenStat version 19 [43].

Correlation coefficients between EPG and life-history metrics were determined using the Data Analysis function in Microsoft Excel (Microsoft, Redmon, WA, USA) to conduct a regression analysis.

## 3. Results

### 3.1. Feeding Behaviour Bioassays

The feeding behaviour of *R. padi* showed a significant difference between endophyte treatments and the control (Nil_E) for 8 of the 102 metrics describing the seven major waveforms (Table 2). For probing, the number of potential drops (pd) was significantly lower for AR37, NEA2 and NEA6 compared to AR1 and Nil_E, with 28–49% fewer events. Likewise, the total time of pd was significantly lower for AR37, NEA2 and NEA6 compared to AR1 and Nil_E, with 31–49% less time spent in pd. Additionally, the percentage of probe time spent in intercellular probing (C) was significantly lower on AR37, NEA2 and NEA6 compared to AR1 and Nil_E, with a 13–38% reduction. Furthermore, the total C time was significantly lower for AR37, NEA2 and NEA6, with 31–39% less time spent in C compared to Nil_E (Table 2).

For feeding, the number of sieve element salivations directly prior to phloem ingestion (E1) was significantly lower for AR37, NEA2 and NEA6 compared to Nil_E, with 32–49% fewer events. The number of phloem sap ingestion (E2) events was significantly lower for AR37, NEA2 and NEA6, with 33–48% fewer events compared to Nil_E. Furthermore, the average duration of the first E1 event was significantly longer for AR37, NEA2 and NEA6 compared to Nil_E, with 41–74% more time spent in the first E1 event. No differences in xylem ingestion (G) or stylet derailment (F) were observed in any treatment.

For the non-probing period (Np), the median Np time was significantly longer for NEA2 and NEA6 compared to Nil_E, with 102 and 120% more time spent in Np, respectively (Table 2).

### 3.2. Life-History

The average mortality of *R. padi* at the nymphal stage was 26% on endophyte-infected plants compared to 8% on the Nil_E control (Table 3). The total nymph mortality was highest on SE and AR37 (33% mortality), followed by NEA6 (25%), AR1 (21%) and NEA2 (17%). While there was no significant difference in overall nymph mortality (*p* = 0.223), there was a trend showing higher nymph mortality on endophyte treatments compared to Nil_E, with the largest difference (0.24) observed between both SE and AR37 and Nil_E. The average nymphal mortality on endophyte treatments was highest at >72 h (15%). The only significant difference in nymph mortality was observed at 48 hr, where SE and AR37 (both 13%) showed significantly (*p* = 0.042) higher mortality than the control (0%) (Table 3).

The average adult mortality was 74% across all endophyte treatments, compared to 92% on the Nil_E control. At 14, 21 and 28 days, there was no significant difference in mortality between treatments. There was a significant (*p* = 0.031) difference in adult mortality at >28 days, with the highest mortality observed on Nil_E (0.75) and the lowest on SE (0.29) (Table 3).

The average fecundity was 16.7 nymphs per adult aphid per day across all endophyte treatments, compared to 21.4 on the Nil_E control (Table 4). Fecundity at 14 days was significantly reduced on all endophyte treatments (*p* = 0.003) compared to the control (Nil_E), with AR37 and NEA2 both exhibiting the strongest reduction in fecundity (both 8.8), followed by SE (9.1), AR1 (10.2) and NEA6 (10.3). Fecundity at 21 days was significantly reduced on all endophyte treatments (*p* = 0.041) compared to the control (20.6), with NEA2 exhibiting the highest reduction in fecundity (14.2), followed by SE (14.3), AR37 (14.6), AR1 (15.2) and NEA6 (16.8). There were no significant differences between the control and any endophyte treatments at 28 days, although the trend continued to show higher fecundity on the control. There were no significant differences between endophyte treatments in any time period, but NEA2 consistently had the lowest fecundity in all time periods (Table 4).

The average intrinsic rate of increase (*r_m_*) was 0.27 across all endophyte treatments compared to 0.33 on the Nil_E control. AR37 exhibited the strongest reduction in *r_m_* (0.24), followed by SE and AR1 (both 0.26), NEA2 (0.28) and NEA6 (0.30). No endophyte treatments exhibited significantly reduced *r_m_* compared to the Nil_E control (*p* = 0.057), whereas all endophyte treatments exhibited lower average *r_m_* than the Nil_E control (Table 4).

### 3.3. Correlation between Feeding Behaviour and Life History

There were strong positive correlations between both the probing metric (percentage of probe time in C, ρ = 0.79) and the feeding metric (no. of E2 events, ρ = 0.72) and fecundity (Table 5). There were no strong positive or negative correlations between any other EPG metric and fecundity. There were no strong positive or negative correlations between nymphal mortality, adult mortality or *r_m_* and any EPG metrics.

## 4. Discussion

### 4.1. Feeding Deterrence

There was a clear endophyte effect on *R. padi* feeding behaviour and its life history. Assessments of the endophyte mode of action using EPG experiments showed that *R. padi* feeding on perennial ryegrass infected with the endophytes AR37, NEA2 or NEA6 spent less time probing (C) and phloem feeding (E2) and more time in the non-probing phase (Np) compared to the Nil_E control, SE and AR1. These results indicate a distinct level of feeding deterrence induced by AR37, NEA2 and NEA6, as they all point to both delayed (increased time to first E2) and reduced (lower number and shorter E2 events) feeding by *R. padi*. Our EPG data suggest a type of resistance that is chemical-based, as aphids were able to reach the phloem and maintain phloem ingestion, albeit reduced in comparison to the Nil_E control. Feeding effects were similar in all cases and thus could not be associated with any specific alkaloid; our EPG data suggest that all alkaloids, regardless of their specific chemistries, are somewhat deterrent, allowing for some phloem ingestion to still occur.

It is also possible that an undetermined compound, other than the known alkaloids, may be contributing to aphid feeding deterrence effects [44,45,46]. A number of additional metabolites have been characterised, such as epoxyjanthitrems, an indole diterpene precursor, a metabolite needed for the production of indole diterpene alkaloids known to exist in *Epichloë* endophytes [47]. This and other precursors could also have important effects on feeding [48,49].

SE produces the same alkaloids as NEA2 but at higher concentrations. SE also produced high *R. padi* mortality in life-history bioassays. Despite this, SE did not deter *R. padi* feeding behaviour, implying that perhaps SE may be producing additional metabolites that suppress any feeding deterrent effects associated with the alkaloids produced. This may be an additional compound produced by the endophyte that masks its unpalatability or a structural change to the alkaloid molecule that alters the palatability while retaining toxic effects.

As endophytes grow in the intercellular plant tissue [50,51], and aphids feed by invading this same intercellular space with their stylets [22,23], it is possible that the presence of endophyte fungal hyphae within the plants may in itself present a physical barrier to feeding by phloem-feeding hemipterans, such as aphids. In addition to chemical-based resistance, we cannot rule out that some degree of physical-based resistance is occurring based on the presence of hyphae; the presence of endophyte hyphae in plant tissue would create a barrier of some sort. The specific morphological characteristics of leaves from *Epichloë*-infected perennial ryegrass plants could be such that aphid stylets’ movement between parenchyma cells and vascular bundles is altered. The examination of plant tissue at the cellular level using the appropriate type of microscopy should reveal whether endophyte hyphae are indeed creating a physical barrier to aphid stylets and how significant this barrier is.

The fact that aphids feeding on endophyte-infected plants exhibited higher non-probing activities by comparison to the Nil_E control indicates some level of deterrence from an external cuticular point of view, i.e., due to morphological changes to the surface of the leaves [52] and/or the presence of volatile organic compounds (VOCs) [9,53]. Studies have shown that endophytic fungi can produce VOCs that affect aphid infestation [54] and act as attractants to aphid predators [55].

### 4.2. Life-History Effects

*Rhopalosiphum padi* fecundity was significantly reduced on all endophyte treatments, particularly in the early to middle stages of reproduction. This indicates not only a reduction in overall aphid fecundity but also a slowing of reproduction, which could lead to slower population growth. Similar results were observed by Meister et al. [56], who found that *R. padi* showed reduced population growth and reduced fecundity when reared on endophyte-infected perennial ryegrass compared to those reared on endophyte-free perennial ryegrass. In another study, *R. padi* showed similarly reduced population growth when reared on tall fescue infected with fungal endophytes [57]. The reduction in overall aphid fecundity, coupled with the reduced feeding seen in this study, indicates that adult *R. padi* may not have been receiving sufficient nutrition to reproduce at their optimal rate, which has been observed in *R. padi* feeding on barley [20]. The reduced fecundity could also reflect direct endophyte toxicity to unborn aphid nymphs, resulting in stillborn nymphs [33]. Bastias et al. [34] also showed that *R. padi* fecundity is negatively affected by the presence of endophytes in annual ryegrass and postulated that this activity was due to direct toxicity. The direct negative effect of *Epichloë* endophytes on aphid fecundity clearly demonstrates its importance in managing aphid populations and broader insect control in pastures.

*Rhopalosiphum padi* nymphs showed higher mortality on endophyte-infected perennial ryegrass compared to control plants at all time periods. While these results were not statistically significant at all time periods, they do show a trend that conforms with previous research with *R. padi* on endophyte-infected grasses [13,34]. Furthermore, nymph mortality was significantly higher on SE and AR37 at 48 hr, similar to the results in Collinson et al. [33], where SE was shown to result in the highest nymph mortality in the Russian wheat aphid *Diuraphis noxia* (Kurdjumov) and the root aphid *Aploneura lentisci* (Passerini). Multiple studies have shown that *R. padi* experiences higher mortality on different varieties of *Epichloë* endophytes, implying that each endophyte delivers a different level of insecticidal activity [13,15,34]. The results of this study also showed a significant difference in the mortality of adult *R. padi* at >28 days, where mortality was significantly higher on control plants, indicating a normal *R. padi* life span, as opposed to the endophyte treatments, where mortality was greatest in the nymphal stage.

## 5. Conclusions

The results of the feeding behaviour and life-history bioassays indicate that *R. padi* was significantly affected by perennial ryegrass infected with endophytes. The effects, however, cannot be directly attributed to any known alkaloid, as there are no known alkaloids shared by all endophytes tested, or to any specific mode of action, as the relative concentrations of metabolites, including precursors, could be affecting aphids’ feeding behaviour and life history. The role of VOCs also needs further consideration. Nevertheless, the feeding deterrence and life-history effects of these endophytes have practical benefits in pasture-based grazing systems, where they can all be used to reduce the population growth of *R. padi* in fields, decreasing aphid pest pressure on pasture systems and thus allowing for a reduction in the use of insecticides. These results show that all of these endophytes can play a pivotal role in the broader pest management scheme for *R. padi* in pasture grazing system production and the importance of studying multitrophic-level interactions between endophytes, their host plant and phytophagous pest insects.

Further work to elucidate the mode of action of endophytes on the feeding and life history of aphids could include the analysis of phloem exudate, i.e., stylectomy, to elucidate metabolite composition, including plant defence response chemicals and alkaloid composition in response to aphid feeding. The morphological analysis of the leaves, cuticular areas and intracellular areas of host plants could also help elucidate endophytes’ mode of action, as could developing in vitro alkaloid toxicity assays using artificial diets to further understand the physiological interactions.

## Figures and Tables

**Table 1:** *Epichloë festucae* var. lolii endophyte strains used in this study and associated alkaloid profiles.

Alkaloid Class	Alkaloid Type	SE	AR1	AR37	NEA2	NEA6
Ergopeptide	Ergovaline	P	*	*	P	P
Polyketide	Peramine	P	P	*	P	P
Indole Diperpene	Epoxyjanthitrems	*	*	P	*	*
Indole Diterpene	Lolitrem B	P	*	*	T	*

The host plant for all endophytes was perennial ryegrass cv. Alto. SE = standard endophyte; AR1, AR37, NEA2, NEA6 = commercial endophytes. P = alkaloid present; * = alkaloid absent; T = alkaloid present only at trace levels.

**Table 2:** Feeding behaviour metrics of *R. padi* on all endophyte and endophyte-free treatments observed using EPG.

Feeding Behaviour		Nil_E (n/a)	SE (LEP)	AR1 (P)	AR37 (E)	NEA2 (LEP)	NEA6 (EP)	LSD	*p*-Value
		24	24	20	24	24	24		
Probing	Number of Potential Drops	99.88	84.00	103.10	71.63	53.00	65.71	0.20	**0.018**
		1.94 ^ab^	1.84 ^abc^	1.96 ^a^	1.70 ^c^	1.68 ^c^	1.72 ^c^		
	Sum of Potential Drop Time (minutes)	8.01	6.80	8.55	5.53	4.38	5.39	0.20	**0.016**
		0.85 ^ab^	0.75 ^abc^	0.88 ^a^	0.60 ^c^	0.61 ^c^	0.64 ^c^		
	Percentage of Probe Time in C	33.01	24.42	25.91	22.60	21.02	20.63	0.22	**0.045**
		1.45 ^a^	1.22 ^b^	1.33 ^ab^	1.14 ^b^	1.21 ^b^	1.14 ^b^		
	Sum of C Time (minutes)	195.07	155.13	149.78	134.96	131.73	118.82	0.21	**0.040**
		2.23 ^a^	2.04 ^ab^	2.11 ^ab^	1.94 ^b^	2.01 ^b^	1.93 ^b^		
Feeding	Number of E1 Events	10.29	7.58	8.65	6.96	5.29	6.63	0.22	**0.042**
		0.96 ^a^	0.75 ^ab^	0.85 ^ab^	0.69 ^b^	0.63 ^b^	0.68 ^b^		
	Number of E2 Events	10.13	7.46	8.60	6.75	5.25	6.46	0.23	**0.045**
		0.95 ^a^	0.74 ^ab^	0.84 ^ab^	0.67 ^b^	0.63 ^b^	0.67 ^b^		
	Average Duration of 1st E1 (minutes)	0.34	0.43	0.42	0.48	0.50	0.59	0.09	**<0.001**
		−0.50 ^a^	−0.38 ^bc^	−0.41 ^ab^	−0.36 ^bcd^	−0.33 ^bcd^	−0.27 ^d^		
Non- probing	Median Np Time (minutes)	8.99	6.86	12.61	10.67	18.17	19.80	0.28	**0.035**
		0.69 ^b^	0.75 ^b^	0.90 ^ab^	0.65 ^b^	0.80 ^ab^	1.07 ^a^		

Data shown as means (BT^x^) Log^10^ scale (^x^ back-transformed to the original scale). *p*-Values and LSDs from ANOVA with logarithmic transformation (base 10). *p*-Values less than 0.05 are presented in bold face. EPG feeding behaviour signals are categorised by the following waveform patterns: Np = non-probing; C = intercellular probing phase; pd = potential drop resulting from cell puncture; E1 = pre-phloem-ingestion salivatory phase; E2 = active phloem ingestion. Endophytes produced the following alkaloids: L = lolitrem B; E = ergovaline; P = peramine; E = Epoxyjanthitrem I. ^a,b,c,d^ treatment means with different superscripts (Duncan’s letter) are significantly different.

**Table 3:** Mortality of *R. padi* on all endophyte and endophyte-free treatments observed at the nymphal stage (24, 48, 72 and >72 h) and the adult stage (14, 21, 28 and >28 days).

Mortality	Nil_E	SE	AR1	AR37	NEA2	NEA6	Average Mortality ^a^	LSD	*p*-Value ^b^
Nymph mortality (24 h)	0/24 (0.00)	1/24 (0.04)	0/24 (0.00)	1/24 (0.04)	3/24 (0.13)	1/24 (0.04)	0.05 (0.097)	0.10	0.232
Nymph mortality (48 h)	0/24 (0.00)	3/24 (0.13)	1/24 (0.04)	3/24 (0.13)	0/24 (0.00)	0/24 (0.00)	0.06 (0.103)	0.10	**0.042**
Nymph mortality (72 h)	0/24 (0.00)	0/24 (0.00)	0/24 (0.00)	0/24 (0.00)	0/24 (0.00)	0/24 (0.00)	0.00 (0.000)	0.00	1.000
Nymph mortality (>72 h)	2/24 (0.08)	4/24 (0.17)	4/24 (0.17)	4/24 (0.17)	1/24 (0.04)	5/24 (0.21)	0.15 (0.161)	0.19	0.477
Nymph mortality (total)	2/24 (0.08)	8/24 (0.33)	5/24 (0.21)	8/24 (0.33)	4/24 (0.17)	6/24 (0.25)	0.26 (0.197)	0.23	0.223
Adult mortality (14 days)	0/24 (0.00)	3/24 (0.13)	5/24 (0.21)	3/24 (0.13)	2/24 (0.08)	3/24 (0.13)	0.13 (0.154)	0.17	0.163
Adult mortality (21 days)	2/24 (0.08)	4/24 (0.17)	2/24 (0.08)	1/24 (0.04)	4/24 (0.17)	3/24 (0.13)	0.12 (0.144)	0.18	0.658
Adult mortality (28 days)	2/24 (0.08)	2/24 (0.08)	3/24 (0.13)	2/24 (0.08)	2/24 (0.08)	0/24 (0.00)	0.08 (0.118)	0.15	0.472
Adult mortality (>28 days)	18/24 (0.75)	7/24 (0.29)	9/24 (0.38)	10/24 (0.42)	12/24 (0.50)	12/24 (0.50)	0.42 (0.222)	0.27	**0.031**
Adult mortality (total)	22/24 (0.92)	16/24 (0.67)	19/24 (0.79)	16/24 (0.67)	20/24 (0.83)	18/24 (0.75)	0.74 (0.206)	0.23	0.223

Results are shown as the number of dead aphids vs. the number tested (calculated proportion in parentheses). ^a^ Average mortality on endophyte treatments (excluding Nil_E) up to 28 days with S.E in parentheses. ^b^ *p*-Values were calculated using a logistic regression analysis. *p*-Values less than 0.05 are presented in bold face. Nil_E = without endophytes; SE = standard endophyte; AR1, AR37, NEA2, NEA6 = commercial endophytes. All bioassays were carried out for 28 days from the moment of birth.

**Table 4:** Fecundity of *R. padi* on all endophyte and endophyte-free treatments.

Fecundity (All Aphids)	Nil_E (*n* = 21)	SE (*n* = 16)	AR1 (*n* = 20)	AR37 (*n* = 18)	NEA2 (*n* = 20)	NEA6 (*n* = 18)	Average Fecundity ^a^	Standard Error	LSD	*p*-Value ^b^
Fecundity (14 days)	14.3	9.1	10.2	8.8	8.8	10.3	9.4	1.6	3.4	**0.003**
Fecundity (21 days)	20.6	14.3	15.2	14.6	14.2	16.8	15	2.4	4.7	**0.041**
Fecundity (28 days)	21.4	15.9	17.1	16.8	15.5	18.0	16.7	2.6	5.2	0.207
Intrinsic rate of increase (*r_m_*)	0.33	0.26	0.26	0.24	0.28	0.30	0.27	0.03	0.06	0.057

Results are shown as means. Only data from aphids that survived to reproduction were analysed. ^a^ Average fecundity per female per day on endophyte treatments (excluding Nil_E) up to 28 days. ^b^ *p*-Values were calculated using a one-way analysis of variance. *p*-Values less than 0.05 are presented in bold face. Nil_E = without endophytes; SE = standard endophyte; AR1, AR37, NEA2, NEA6 = commercial endophytes.

**Table 5:** Coefficients of correlation between significant EPG metrics and life-history metrics of *R. padi*.

EPG Metric	Nymph Mortality	Adult Mortality	Fecundity	*r_m_*
PROBING				
Number of Potential Drops	−0.25	0.25	*0.50*	0.19
Sum of Potential Drop Time (minutes)	−0.28	0.28	0.49	0.20
Percentage of Probe Time in C	−0.60	*0.60*	**0.79**	0.53
Sum of C Time (minutes)	*−0.55*	*0.55*	*0.69*	0.46
FEEDING				
Number of E1 Events	−0.31	0.31	*0.60*	0.31
Number of E2 Events	−0.44	0.44	**0.72**	0.44
Average Duration of 1st E1 (minutes)	0.32	−0.32	−0.48	−0.12
NON-PROBING				
Median Np Time (minutes)	−0.19	0.19	−0.07	0.22

EPG = electrical penetration graph. Correlations shown as coefficients between −1 and +1, where −1 represents a perfect negative correlation, 0 represents no correlation, and +1 represents a perfect positive correlation. Coefficients greater than +/−0.7 (in bold face) are considered strong correlations, and coefficients between +/−0.5 and +/−0.7 (in italics) are considered correlations.

## Data Availability

All relevant data are within the manuscript. The COI molecular data are available from the NCBI database (accession number MT119781).
